# Deep Learning Algorithm for Online College Physical Education Teaching with Flipping Classroom

**DOI:** 10.1155/2022/8742661

**Published:** 2022-08-24

**Authors:** Zhengqiang Chen

**Affiliations:** Basic Teaching Department, Zhejiang Tongji Vocational College of Science and Technology, Hangzhou 311231, China

## Abstract

In view of the inability to accurately analyze the application of deep learning in college physical education teaching design from the perspective of flipping classroom, this paper puts forward an improved deep learning method based on the integration of flipping classroom vision and deep learning, which can reduce the design ability of physical education teaching design in college physical education teaching design and improve the level of college physical education teaching design. Firstly, the initial data set is established by using the theory of flipping classroom horizon, so that the data meet the requirements of normal distribution and reduce the differences between teaching data; Then, the physical education teaching design is divided into different subdesigns by using the theory of flipping classroom horizon. Find the best design result in this domain in each subinstructional design; Finally, under the guidance of the theory of flipping classroom horizon, each subdesign realizes the optimal allocation of teaching resources. MATLAB simulation shows that under the conditions of initial design scheme and teaching resources setting, the improved deep learning method can improve the accuracy of physical education teaching design and shorten the convergence time of design, which is superior to the original deep learning method. Therefore, the deep learning method is used to analyze the instructional design of college physical education, which has a good design effect and is suitable for the instructional design of college physical education.

## 1. Introduction

With the continuous improvement of physical education teaching level, the requirements of instructional design are becoming more and more strict, and it presents a complicated direction of development Yu, and Zhu [1], so it is particularly important to study physical education instructional design under complex conditions. The key to the allocation of teaching resources in physical education teaching design is to judge the types of resources and design requirements Yin [2]. The theory of flipping classroom horizon can not only adjust the dynamic relationship between teaching content and resources but also solve the selection of physical education teaching design under multiple horizons, which is the main theory of physical education teaching design at present Xiao et al. [3]. Flipping the classroom horizon can make teachers realize the significance of preview and its importance to physical education. Flipping classroom takes students as the main core, which can give full play to students' initiative and change the center of traditional teaching. Relatively speaking, flipping the classroom can distinguish the primary and secondary of teaching, so that students can study deeply according to their own interests. Therefore, flipping classroom plays a very important role in physical education teaching design. Literature research shows that the theory of flipping classroom horizon classifies physical education teaching design, shortens the design time and improves the overall level of design. However, in the process of design classification by flipping classroom horizon theory, the complexity of design will affect the judgment of conditions and the final design result Webb et al. [4]. Some scholars have put forward the deep learning method. Although the deep learning method can realize the selection of design schemes, it is suitable for the conditions with less design requirements. Although there are many researches on flipping classroom by scholars at home and abroad, there are few researches on other methods from the perspective of flipping classroom, and deep learning methods can better promote the improvement of physical education teaching design level. At present, scholars at home and abroad have little research on deep learning from the perspective of flipped classroom, especially the actual case analysis. Under changeable and complex conditions, the overall design ability of the design scheme is greatly reduced Wang and Wang [5]. Therefore, some scholars put forward the theory of flipping classroom horizon and improving deep learning, as shown in Table 1.

**Figure 1:**
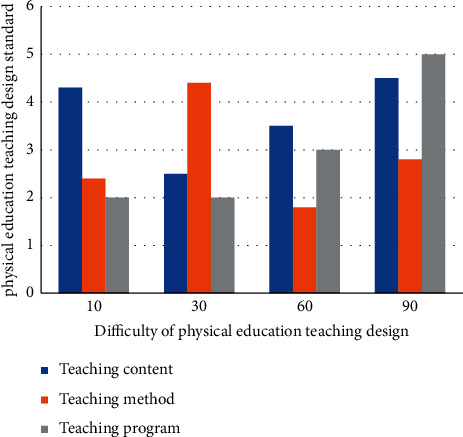
Relationship between difficulty and standard in physical education teaching.

It can be seen from Table 1 that the complexity of college physical education teaching design is high from the perspective of flipping classroom. However, to a certain extent, it also shows the need of intelligent algorithm for college physical education teaching design. Among them, shooting, martial arts, 100 meters, and high jump need intelligent algorithms to achieve intelligent management and analysis. At the same time, some scholars have studied the integration of deep learning method and flipped classroom horizon theory, and found that the flipped classroom horizon theory can reduce the complex design requirements and improve the reliability of physical education teaching design Umezawa et al. [6]. Based on this, this paper puts forward an improved deep learning method based on the integration of deep learning and flipping classroom horizon theory, designs physical education under complex conditions, and verifies the effectiveness of the design. At present, there is a dynamic relationship between design difficulty and standard in college physical education teaching design, as shown in Figure 1.

As can be seen from Figure 1, there is a positive correlation between physical education teaching design and standards, so it is necessary to strengthen the difficulty analysis of physical education teaching design to meet the design standards and requirements Trpkovska et al. [7].

To sum up, there are many researches on flipping classroom horizon, which can provide support for deep learning and better promote the improvement of college physical education teaching design level. At the same time, the integration of deep learning and physical education teaching design requires more reasonable adjustment. Flipping the classroom can provide preview in the early stage and discussion in the later stage, and realize a series of teaching optimization. At present, the research of physical education teaching design is developing in the direction of intelligence, and turning over the classroom horizon lays the foundation for this development, which is also the focus of future research.

## 2. Related Concepts

### 2.1. Flip the Classroom Horizon Theory

The theory of flipping classroom horizon was first put forward by Eric Mazur in 1990s, which is based on peer teaching method. Compared with the traditional teaching mode, the theory of flipping classroom horizon not only pays attention to information transmission, but also deepens the understanding of knowledge Tang, and Wang [8]. The theory of flipping classroom horizon requires students to teach themselves the course content after class and interact in the form of “asking questions-thinking-answering” in class. According to the proportion of students' correct answers, teachers should adjust their teaching contents J. Song, and Kapur [9], so as to deepen students' understanding of knowledge and make them understand the key points and difficulties of teaching. However, flipping classroom is the best combination put forward from the perspectives of teachers, teaching, and students, which has the characteristics of sparsity and integration, and can be designed dynamically in physical education. The theory of flipping classroom horizon needs to construct core functions and analyze the experience and requirements in physical education teaching design in order to improve the accuracy of the design scheme Soler et al. [10].

**Theorem 1 .**

Assuming a collection of physical education instructional design schemes, *T*=(*|d*_*i*_∀ *b*_*i*_*i*=any) arbitrary physical education instructional design *q*_*i*_ ∈ [−*∞*, +*∞*], innovative instructional design *b*_*i*_ ∈ [1,100], and adopted design methods *K*(*d*_*i*_⇒*q*_*i*_⇔*b*_*i*_). The resource allocation function matches the teaching resources with the educational requirements *L*(*x*_*i*_⇔*y*_*i*_ ⇐ *z*_*i*_), and the sports design scheme makes the scheme optimal *Y*_*f*_. The calculation is shown in formula.

(1) $$\begin{array}{c} Y_{f}=w\times f\left(d_{i}\Rightarrow q_{i}\Leftrightarrow b_{i}\right)⇐\lambda . \end{array}$$

Among them, *w* is the allocation condition of different resources and *λ* is the teaching resource. When ∑*Y*_*f*_ ≈ *∞*, the number of physical education teaching design classifications was the largest, the classification interval was the largest, and the design was more difficult; when *w*≃1, the design was the most innovative.

**Theorem 2 .**

If the deviation of physical education teaching design *ξ*_*i*_ is between [−1, 1], it means that physical education teaching design is reasonably optimized, otherwise, it should be re-optimized, and the optimal scheme *Y*_*f*_ calculation is shown in formula.

(2) $$\begin{array}{c} \left\{\begin{array}{c} \min Y_{f}=w\cdot K\left(d_{i},q_{i},b_{i}\right)\;\forall C\sum \xi_{i},\\ w\cdot K\left(d_{i},q_{i},b_{i}\right)≃1. \end{array}\right. \end{array}$$

Among them, *C* is deviation, which reflects the deviation between physical education teaching design and design conditions Sofya, and Sahara [11].

**Theorem 3 .**

If the resource allocation function *K*(*d*_*i*_⇒*q*_*i*_⇔*b*_*i*_)=∑*φ*(*d*_*i*_)⇔∑*φ*(*q*_*i*_), the matching function *L*(*x*_*i*_, *y*_*i*_, *z*_*i*_) is calculated as shown in the following formula:

(3) $$\begin{array}{c} \left\{\begin{array}{c} \max L\left(a\right)=\sum a_{i}\Leftrightarrow \sum a_{ij}q_{ij},\\ \sum a_{i}=dx_{i}\Leftrightarrow q_{i}. \end{array}\right. \end{array}$$

Among them, flipping the deviation coefficient *C* in the theory of classroom horizon is the key to the implementation of deep learning method Shim et al. [12].

### 2.2. Improve the Deep Learning Method

#### 2.2.1. Deep Learning Can Realize the Optimization of Large-Scale Physical Education Teaching Design by Simulating Artificial Behavior, Including Leading, Assisting, and Adjusting

During initialization, the number of PE instructional design and instructional design innovation schemes is the same, and the matching of different PE instructional designs represents the optimal solution. Firstly, the initialization of physical education teaching design and physical education teaching design are randomly generated, and the physical education teaching design is judged near the scheme with better fitness scheme Luo and Zhu [13], and the “poor” scheme is eliminated by comparison, about 1/2 of the number; Then, the auxiliary scheme uses roulette strategy to judge the best scheme, gives corresponding weights, and carries out greedy judgment around the optimal scheme to generate a 1/2 scheme. Finally, the physical education teaching design that does not conform to the teaching resources needs to be abandoned, and the physical education teaching design should be judged in other directions [14].

Assuming that the initial number of physical education teaching design and innovative schemes is *n*, and the random matching of physical education teaching design is *L*=(*x*_*i*_, *y*_*i*_, *z*_*i*_), *x*_*i*_, *y*_*i*_ represent plane coordinates and *z*_*i*_ represents difficulty, then the initial matching calculation of physical education teaching design is shown in formula:

(4) $$\begin{array}{c} L_{i}\left(x_{i},y_{i},z_{i}\right)=w\cdot K\left(d_{i}\Rightarrow q_{i}⇐b_{i}\right)K\left\{\left(x_{j\max},y_{j\min},z_{j\min}\right)⟶L\left(0,1\right)\right., \end{array}$$

where, *x*_*i*_, *y*_*i*_, and *z*_*i*_ are any matching resources *x*_*j*max_ is the scheme with the greatest complexity, *y*_*j*min_ and *z*_*j*min_ the sum is the scheme with the smallest complexity. *L*(0,1) is a random number in the range of [0, 1].

The improvement scheme is to randomly allocate physical education teaching resources and make cross judgment among resources to realize the renewal of physical education teaching design. Under the constraint of fitness, the optimal resource matching is obtained by using the most complex conditions, and the calculation is shown in formula:

(5) $$\begin{array}{c} \Delta L_{i}\left(x_{i},y_{i},z_{i}\right)=w\cdot K\left(d_{i},q_{i},b_{i}\right)\Leftrightarrow \int \varphi_{ijz}\frac{\left(\Delta x_{io}\Rightarrow \Delta y_{io}⇐\Delta z_{io}\right)}{\sum \lambda \cdot K\left(d_{i}\Rightarrow q_{i}⇐b_{i}|\right)}. \end{array}$$

Among them, *o*, *i* ∈ [0, *n*], *φ*_*ijz*_ ∈ (−2,1).

The auxiliary scheme *p*_*i*_ is to adjust the physical education teaching design by probability method, and judge the neighborhood of the better physical education teaching design to obtain the optimal design result, which is calculated as shown in formula:

(6) $$\begin{array}{c} p_{i}=\frac{K\left(b_{i}\right)}{\sum_{i,j,k}^{n}\left[K\left(\Delta d_{io}\Rightarrow \Delta q_{io}⇐\Delta b_{i}\right)\right]}. \end{array}$$

Among them, *F*(·) is a moderate function with different complexity.

If the physical education teaching design has not got the optimal solution after circular adjustment, the complexity will be reduced, and then the physical education teaching design will be judged.

#### 2.2.2. Dynamic Optimization of Physical Education Teaching Design

In the preliminary analysis, physical education teaching design can not guarantee the whole design, which may increase the subjectivity of the design and reduce the overall effect of the design. Therefore, in the process of physical education teaching design, we should try our best to expand the use of teaching resources and constantly adjust teaching resources. Some scholars adjust teaching resources *ρ* linearly to reduce the randomness in the selection of teaching resources, but there is still “partial subjectivity” in the selection of teaching resources. In order to make up for the above shortcomings, the adjustment factor *ν* is introduced in this paper, and the calculation is as shown in formula:

(7) $$\begin{array}{c} \rho =\min\sum \Delta \nu_{i}\Rightarrow \;\log\;e^{-F\left(x_{i},y_{i},z_{i}\right)/\sum F\left(x_{i},y_{i},z_{i}\right)}. \end{array}$$

Among them, Δ*ν*_*i*_ is *i* times dynamic adjustment and *F*(*x*_*i*_⇒*y*_*i*_ ⇐ *z*_*i*_) is *i* times update function Gayef [15]. The matching function between teaching resources and teaching design can be obtained from formulas (6) and (7), and the calculation is shown in formula

(8) $$\begin{array}{c} \Delta L\left(x_{i},y_{i},z_{i}\right)=w\cdot K\left(d_{i},q_{i},b_{i}\right)+\nu_{ijz}K\left(\Delta d_{ik},\Delta q_{ik},\Delta b_{ik}\right). \end{array}$$

**Figure 2:**
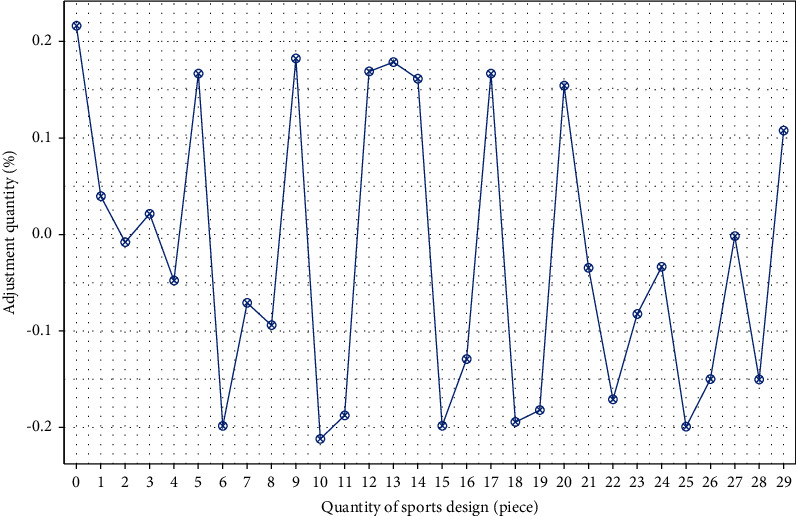
Dynamic adjustment results of physical education teaching resources.

It can be seen from formula (8) that the complexity of (*F*(*x*_*i*_, *y*_*i*_, *z*_*i*_)/∑*F*(*x*_*i*_, *y*_*i*_, *z*_*i*_))=1 and *ν*=1 is the smallest and the complexity of time and time is the largest in preliminary analysis. In order to reduce the “subjectivity” in instructional design, we should keep the diversity of teaching resources. In the final judgment of instructional design (*F*(*x*_*i*_, *y*_*i*_, *z*_*i*_)/∑*F*(*x*_*i*_, *y*_*i*_, *z*_*i*_))=1, *ν* harmony plays an important role, which can not only reduce the complexity of design but also improve the processing ability of design resources and play the role of deep learning method. The result is shown in Figure 2.

As can be seen from Figure 2, the dynamic adjustment of physical education teaching design can accurately carry out the overall design, and the teaching design and teaching resources can be matched. This shows that the dynamic adjustment of physical education teaching design can meet different complexities and improve the effect of teaching design Fang and Jiang [16]. At the same time, the number of physical education teaching designs is relatively uniform, and the adjustment degree is relatively average, which shows that deep learning can achieve the balance of physical education teaching design. Under the condition of ensuring the requirements of physical education teaching design, the analysis of physical education teaching should be carried out to the greatest extent, and the adjustment level of teaching design should be ensured.

#### 2.2.3. Introduction of Flip Classroom Horizon Factor

When a certain physical education teaching design has been adjusted many times and meets the best standard, leading to flip the classroom horizon factor can make the teaching design more in line with “asking-thinking-answering” and get a new solution. Because of the randomness of deep learning method, “subjectivity” has great influence, which will reduce the objectivity of design and do not meet the relevant requirements. In order to reduce the probability of “subjectivity” in physical education teaching design and meet the requirements of different complexities. The “flip class horizon factor” can be introduced by probability density calculation, which is shown in formula:

(9) $$\begin{array}{c} L\left(x_{i}\right)=\frac{\underset{\delta x⟶0}{\lim}\left(F\left(x_{i},y_{i},z_{i}\right)/\sum F\left(x_{i}\Rightarrow y_{i}⇐z_{i}\right)\right)}{\pi \sum_{i,k=1}^{+\infty }{\left|K\left(\Delta x_{ik}\Rightarrow \Delta y_{ik}⇐\Delta z_{ik}\right)\right|}^{2}}. \end{array}$$

If the educational design meets the sum of $\underset{x⟶0}{\lim}\left(F\left(x_{i},y_{i},z_{i}\right)/\sum F\left(x_{i},y_{i},z_{i}\right)\right)≃1$ and *F*(*x*_*i*_⇒*y*_*i*_ ⇐ *z*_*i*_) = 1 it shows that the physical education teaching design is the best, otherwise the design does not meet the requirements Fan, and Meng [17].

### 2.3. Analysis of Physical Education Teaching Design from the Perspective of Flipping Classroom

#### 2.3.1. Optimization Model of Physical Education Teaching Design from the Perspective of Flipping Classroom

Rationality judgment of physical education teaching design from the perspective of flipping classroom Collins [18], it is the main index to measure the deep learning method. From formula (8), it can be seen that in the early stage of physical education teaching design, great attention is paid to the overall utilization of teaching resources, and in the later stage of design, attention is paid to the utilization of local teaching resources, so different design strategies should be adopted in different stages of physical education teaching design. At present, besides the improved deep learning method proposed in this paper, there are other dynamic optimization models.

(1) The adjustment strategy of specific instructional design is calculated as shown in formula:
  (10) $$\begin{array}{c} \Delta L_{i}\left(x_{i}\right)=\sum_{t}\Delta L_{i-1}\left(x_{i}\Rightarrow y_{i}⇐z_{i-1}\right)\forall \left[p\cdot K\left(\Delta d_{ik},\Delta q_{ik},\Delta b_{i-1k}\right)\right]. \end{array}$$
(2) The adjustment strategy of the whole instructional design is calculated as shown in formula:
  (11) $$\begin{array}{c} \Delta L_{i}\left(x_{i}\right)=\sum_{t}\Delta L_{i-1}\left(x_{i-1}⟶y_{i-1}\leftarrow z_{i-1}\right)\forall \left[g\cdot \max K\left(\Delta d_{i-1k},\Delta q_{i-1k},\Delta b_{i-1k}\right)\right]. \end{array}$$
(3) The adjustment strategy of teaching resources is calculated as shown in formula:
  (12) $$\begin{array}{c} \Delta L_{i}\left(x_{i}\right)=\sum_{i=1,t}^{n/2}\Delta L_{i-1}\left(x_{i}\Rightarrow y_{i}⇐z_{i-1}\right)\forall \left[\max K\left(g\right)\forall \max K\left(p\right)\right]. \end{array}$$
(4) The adjustment strategy of multiview instructional design is calculated as shown in formula:
  (13) $$\begin{array}{c} \Delta L_{i}\left(x_{i}\right)=\sum_{t}\Delta L_{i-1}\left(x_{i-1}⟶y_{i-1}\leftarrow z_{i-1}\right)\cdot F\left(x_{i-1},y_{i-1},z_{i-1}\right)\forall K\left(\Delta d_{i-1k}⟶\Delta q_{i-1k}\leftarrow \Delta b_{i-1k}\right). \end{array}$$

Among them, *T* is the time designed for physical education.

In this paper, the deep learning method is improved in two aspects: on the one hand, the physical education teaching design is constantly adjusted. Under the constraint of weight and threshold, we choose randomly from five strategies and complete many adjustments of physical education teaching design. In the later period of physical education teaching design, the use of physical education teaching resources is gradually reduced, and small-scale design adjustment is carried out to keep the diversity of design and improve the overall design ability. On the other hand, we should balance the relationship between physical education teaching design and resources, and integrate renewal coefficient, Δ*ν*_*i*_ moderate function *F*(*x*_*i*_, *y*_*i*_, *z*_*i*_) and Lagrangian multiplier function to carry out physical education teaching design more quickly.

#### 2.3.2. Complexity Adjustment of Physical Education Teaching Design from the Perspective of Flipping Classroom

The complexity of physical education teaching design is the key to realize dynamic optimization. This paper based on the complexity optimization of physical education teaching design from the perspective of flipped classroom can further improve the design effect. Different physical education teaching design complexities adopt different optimization strategies. The physical education teaching design is randomly divided into five subclasses, each subclass represents the scope of physical education teaching design in No. 1 Middle School. In each iteration process, different schemes will be randomly selected from subclasses. After each subclass is adjusted, the fitness and resource utilization of physical education teaching design in different sub-classes is compared, and the overall optimal matching design is recorded; other subclasses gather to the optimal matching design to improve the level of the optimal physical education teaching design.

### 2.4. Flip the Steps of Physical Education Teaching Design from the Perspective of Classroom

**Figure 3:**
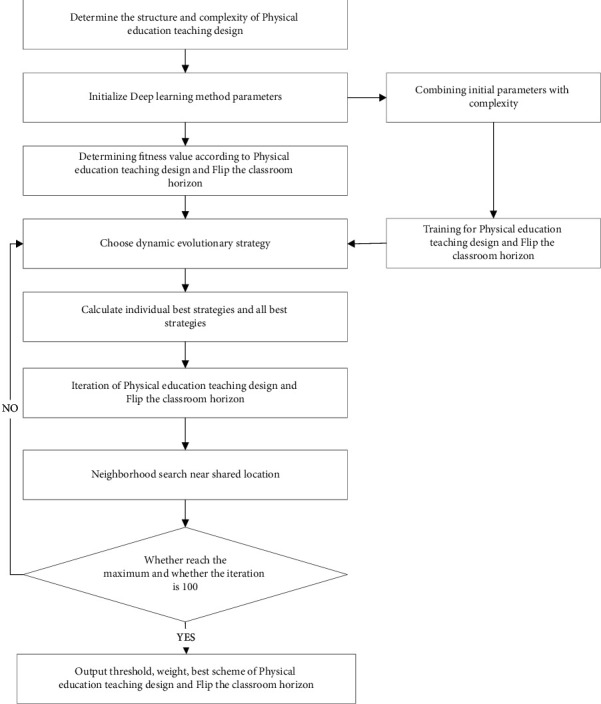
Implementation flow of improved deep learning method.

The basic idea of deep learning method from the perspective of flipping classroom is to use various strategies to optimize physical education teaching design, improve the utilization rate of teaching resources, obtain the overall optimal design scheme, and meet the requirements of design complexity. The implementation steps of physical education teaching design from the perspective of flipping classroom in this paper are shown in the Figure 3:

  Step 1: Determine the structure and complexity of physical education teaching design. According to the actual design requirements, determine the steps of physical education teaching design, generally speaking, the complexity of physical education teaching design.
  Step 2: Initialize the physical education teaching design. According to the relevant instructional design parameters, the physical education instructional design is initialized. The number of physical education teaching designs *n* = 120, and the number of iterations *m* = 100.
  Step 3: Determine the fitness function. Using the theory of flipping classroom horizon, the physical education teaching design scheme is randomly produced, and combined with teaching materials, the initial weight *w*and complexity *λ* are obtained, that is *w*=0.32, *λ*=0.62. Through formulas (3)–(7), the physical education teaching design is improved, and the fitness scheme of each physical education teaching design is obtained.
  Step 4: The whole and local optimal matching of instructional design. The initial physical education teaching design is divided into five subclasses, and the fitness degree is obtained, and the corresponding local matching and overall optimal matching are calculated.
  Step 5: Update iteration of physical education teaching design. According to the requirements of physical education teaching design, the five subclasses dynamically adjust the flipping classroom horizon factor, randomly select strategies from the five subclasses, and integrate the flipping classroom horizon factor C according to formulas (2) and (7).
  Step 6: Dynamic optimization of each subclass. After optimizing the local physical education instructional design, the overall optimal design is selected, and the instructional design is shared with other subclasses, and the neighborhood optimal instructional design scheme is adjusted.
  Step 7: Judge whether the physical education teaching design reaches the maximum iteration value *M*. If it is not reached, repeat steps 1–5, otherwise stop iterative calculation and return to the best physical education teaching design and complexity.

## 3. Empirical Case Analysis

### 3.1. Model Performance Analysis

In order to further judge and improve the deep learning method, four tests were conducted, namely, the utilization rate of teaching resources, the satisfaction rate of students, the feedback rate of teaching, and the thinking situation of students. The test process is as follows.

(a) The utilization function of teaching resources is calculated as shown in formula:
  (14) $$\begin{array}{c} f\left(x\right)=\sum_{i=1}\frac{x_{i}^{2}}{10}\cdot \;\cos\left(2\pi x_{i}\right)+\xi^{\prime }. \end{array}$$
(b) The student satisfaction rate function is calculated as shown in formula:
  (15) $$\begin{array}{c} f\left(x\right)=\left|\sum_{i=1}x_{i}^{2}\right|. \end{array}$$
(c) The feedback rate function of teaching is calculated as shown in formula:
  (16) fx=e1/n∑i=1cos2πxi,1−limx⟶0exi/51/n.
(d) The student's thinking function is calculated as shown in formula:
  (17) $$\begin{array}{c} f\left(x\right)=\sum_{i=1}\frac{x_{i}^{2}}{100}\pm \prod_{i=1}\left|\cos\left(\frac{x_{i}}{\sqrt{i}}\right)\right|. \end{array}$$

In this paper, the parameters of physical education teaching design are set: the total number of physical education teaching resources is 50, the iteration times are 120, and each teaching design is analyzed independently. The results of the four test functions are shown in Table 2.

**Figure 4:**
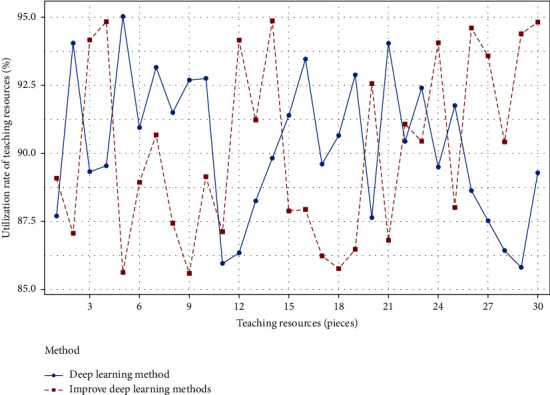
Convergence curve of the best scheme for selecting the test function of teaching resource utilization rate.

It can be seen from Table 2 that the improved deep learning method is superior to the deep learning method, and its overall optimal design and theoretical optimal design Chew et al. [19]. The maximum value of improved deep learning is less than deep learning, while the minimum value is greater than deep learning, and the global design value of improved deep learning is greater than deep learning, so the improved deep learning method is better, the overall result is more reasonable, and it can promote the development of physical education teaching design. Moreover, the analysis scope, resource matching rate and analysis error of the improved deep learning method are smaller than those of the deep learning method. In order to reflect the test results of the test function in 4 more intuitively, the following convergence curves are given, as shown in Figure 4.

**Figure 5:**
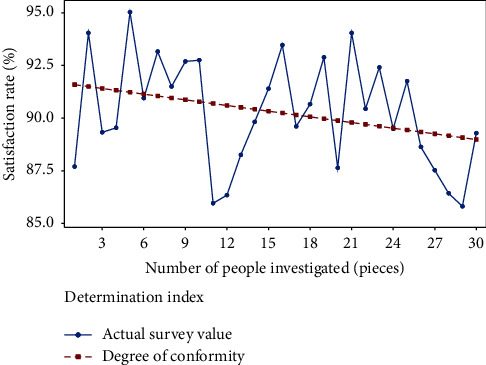
Satisfaction rate test of improved deep learning methods.

It can be seen from Figure 4 that the resource utilization rate of the improved deep learning method is higher than that of the deep learning method, and the change range is relatively large, which makes the processing more difficult. In Figure 4, the number of inflection points of improving deep learning is less than that of deep learning, and the method of improving deep learning is more stable, which makes the college physical education teaching design more scientific and can comprehensively improve the level of physical education teaching design. The result is shown in Figure 5.

It can be seen from Figure 5 that the design satisfaction rate of the improved deep learning method is concentrated between 40% and 90%, which shows that the overall effect of the satisfaction rate is high and meets the requirements of physical education teaching design.

**Figure 6:**
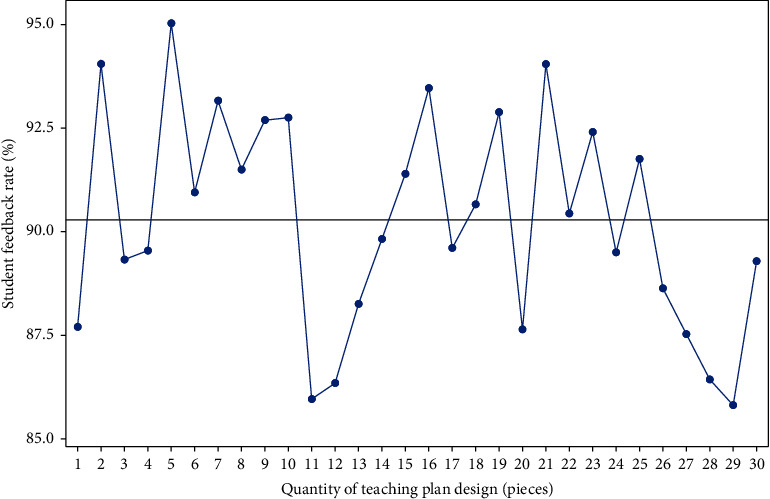
feedback rate test of teaching with different functions.

The data in Figure 5 are scattered, but the overall distribution is relatively concentrated. Therefore, improving deep learning can optimize the physical education teaching design, and the optimization effect is relatively stable. In the optimization process, the results show scattered distribution, which meets the requirements of objective normal distribution. The result is shown in Figure 6.

**Figure 7:**
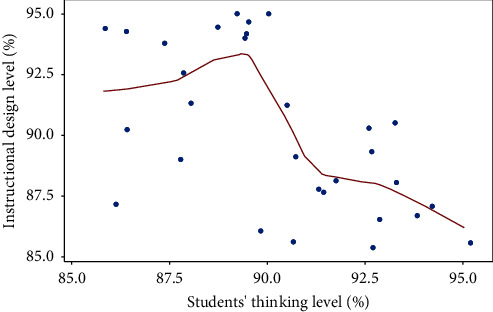
Students' thinking levels of different functions.

It can be seen from Figure 6 that the feedback rate of the improved deep learning method is significantly higher than that of the deep learning method, which shows that flipping the classroom horizon can improve the feedback rate of students and the level of physical education teaching design. The result is shown in Figure 7.

It can be seen from Figures 4–7 that the improved deep learning method has faster calculation speed and better stability, which is superior to the deep learning method. Therefore, the improved deep learning method is suitable for the analysis of college physical education teaching design, and the design process is more stable.

### 3.2. Design and Treatment of Actual Cases

In this paper, football, tennis, swimming, high jump, and other types are selected as research objects, and the collection time was from January 2021 to January 2022. A total of 402 lesson plans were collected, and the class hours were 1023 hours. According to the requirements of the Ministry of Education for physical education, the above-mentioned physical education design is divided into four categories: question-thinking, thinking-answering, question-thinking-answering, question-thinking-answering-feedback. The results are shown in Table 3. This paper verifies the accuracy of the analysis results according to theoretical judgment and actual detection methods. In order to avoid too many subjective factors in physical education teaching design, DETEL function is called to eliminate the design, which makes the physical education teaching design meet the relevant conditions. The results are shown in Table 3.

The physical education teaching design is trained by dichotomy, the first half is the research object, the second half is the analysis object, and the overall results are compared.

### 3.3. Final Research Results

**Figure 8:**
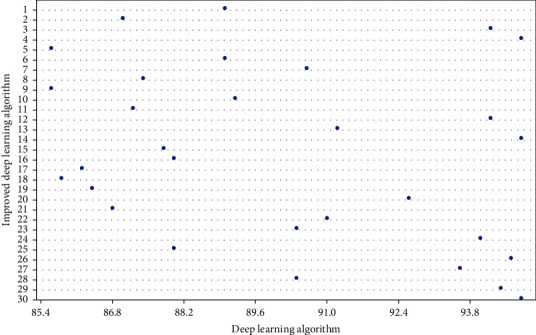
Classification results of complex physical education teaching design.

According to the research conditions of physical education teaching design, the structure of physical education teaching design collection is determined as: structural design-semi-structural design-non-structural design, which meets the analysis requirements of improving deep learning methods. In this paper, the classification results of physical education teaching design by improving deep learning method are proposed, as shown in Figure 8.

Through comparative analysis, we can see that the classification of the improved deep learning method is discrete, which is closer to the actual situation, while the classification of the deep learning method is concentrated and cannot meet the needs of actual classification. In addition, the classification of improved deep learning method is not affected by complexity, while the classification of deep learning method is obviously affected by complexity, and becomes more concentrated with the increase in complexity.

**Figure 9:**
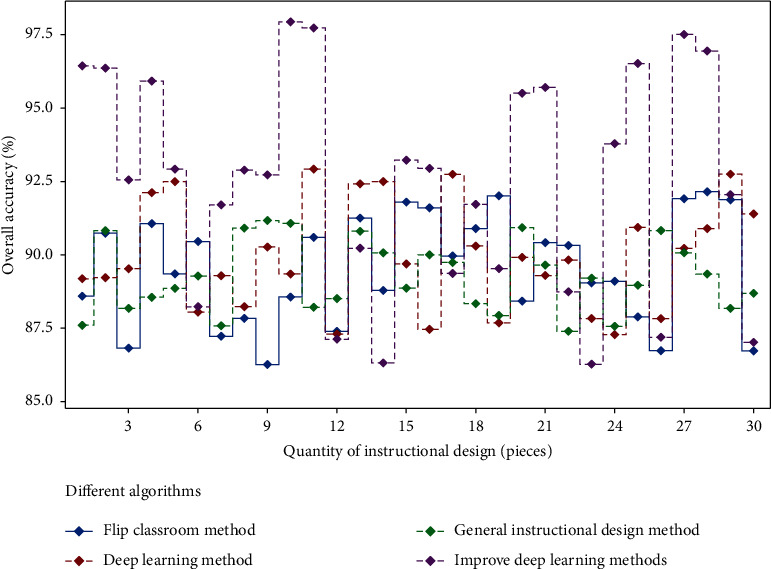
Comparison of overall accuracy of different methods.

The reason is that the improvement of deep learning method adds the theory of changing classroom horizon, and establishes a mapping among questioning, thinking, answering, and feedback, which makes the physical education teaching design more in line with the requirements. At the same time, fitness function is used to adjust physical education teaching design. In order to further prove the effectiveness of the model proposed in this paper, other comparative models are introduced for comparative analysis, and the results are shown in Figure 9.

It can be seen from the above figure that the fitness value of the improved deep learning method is the highest and reaches the limit at the earliest. Under the same complexity, the improved deep learning method has higher stability; The second is the deep learning method. The reason is that the theory of flipping classroom horizon reduces the influence of complexity on physical education teaching design; Different optimization strategies improve the accuracy of design, which is consistent with related research [19]. From the aspect of physical education teaching design types, this paper analyzes the accuracy of different deep learning methods, and the results are shown in Table 4.

It can be seen from the above table that improving the deep learning method can not only improve the efficiency of physical education teaching design but also keep the accuracy unchanged with the change of design type. The main reason is that the dynamic analysis of physical education teaching design from the perspective of classroom makes the time of physical education teaching design shorter and more flexible. Therefore, flipping the classroom horizon theory can not only reduce the impact of complexity on the results but also meet the requirements of different design types.

## 4. Conclusion

This paper puts forward an improved deep learning method based on the theory of flipping classroom horizon, which optimizes the physical education teaching design by setting teaching resources, weights, and designing strategies. The improved deep learning method constructed in this paper can classify the physical education teaching design discretely which makes the physical education teaching design more in line with the actual requirements. MATLAB simulation results show that the deep learning method constructed in this paper has high design accuracy, the accuracy is concentrated in 90∼98%, the single design quantity is relatively stable, accounting for about 3∼4% of the total, the overall convergence is relatively short, and the convergence is carried out between 10 and 20 iterations, which can deal with physical education teaching design with complexity less than 45%, and the global optimal value is 2∼3, the results are consistent with related studies. However, in the analysis of complexity, there are still deficiencies, future research in-depth analysis, to further improve the requirements of college physical education teaching design.

## Figures and Tables

**Table 1:** Complexity of physical education teaching design requirements from the perspective of flipping classroom.

Classification	Instructional design in colleges and universities		Physical education teaching design	
	Technical production	Content modification	Production innovation	Policy selection
Football	4.12	20.62	36.08	43.30
Tennis	38.14	42.27	21.65	18.56
Volleyball	9.28	10.31	29.90	15.46
Swimming	6.19	9.28	17.53	4.12
Yoga	7.22	22.68	36.08	23.71
Fitness	23.71	23.71	25.77	30.93
Martial arts	11.34	45.36	8.25	12.37
100 meters	41.24	44.33	20.62	30.93
500 meters	7.22	16.49	43.30	4.12
High jump	29.90	28.87	15.46	6.19
Long jump	15.46	20.62	10.31	22.68
Shot put	15.46	44.33	42.27	15.46
Javelin	18.56	12.37	39.18	8.25

**Table 2:** Results of different test functions.

Function	Method	Minimum value	Maximum value	SD	Global optimal design	Local optimal design
Utilization rate of teaching resources	Deep learning method	38.14	48.45	3.72E − 05	97.94	1
	Improve deep learning methods	35.05	46.39	5.18E − 05	96.91	3

Student satisfaction rate	Deep learning method	47.42	45.36	4.73E − 05	96.91	2
	Improve deep learning methods	40.21	55.67	5.07E − 05	97.94	1

Feedback rate of teaching	Deep learning method	47.42	51.55	4.17E − 05	97.94	2
	Improve deep learning methods	31.96	50.52	3.94E − 05	98.97	3

Students' thinking situation	Deep learning method	40.21	50.52	4.39E − 05	97.94	1
	Improve deep learning methods	45.36	56.70	4.28E − 05	98.97	2

**Table 3:** Types and proportion of physical education teaching design.

Design type	Quantity of physical education teaching design (pieces)	Proportion (%)
Ask questions-think	99	24.63
Ask-think-answer	110	27.36
Ask-think-answer	91	22.64
Ask-think-answer-feedback	102	25.37

**Table 4:** Overall accuracy of different design types.

Design type	Improve deep learning methods	Deep learning method	Flip classroom method	General instructional design method
Ask questions-think	93.94	91.97	91.97	91.90
Ask-think-answer	97.91	96.90	92.91	91.91
Ask-think-answer	98.92	98.92	92.97	91.92
Ask-think-answer-feedback	98.93	97.93	97.92	92.91

## Data Availability

The experimental data used to support the findings of this study are available from the corresponding author upon request.
